# The Prevention of Shock and Vomiting from Chloroform Anesthesia

**Published:** 1912-07-15

**Authors:** E. Stuver


### THE PREVENTION OF SHOCK AND VOMITING FROM CHLOROFORM ANESTHESIA.

BY E. STUVER, M.D.

March 24, 1899, I resorted for the first time to the use of cocaine, painted on the mucous membrane, to prevent vomiting during chloroform anesthesia. It acted very nicely, preventing the vomiting in nearly every instance, although some of my patients had nearly died from vomiting in previous operations. My opportunities were quite limited, but, in a short article in the July, 1903, issue of *The Denver Medical Times*, I called attention to this use of cocaine as follows:

"Meyer and Prebrium, in 1873, showed that gastric irritation inhibits the heart's action. Guirni (see J. *A*. *M*. *A*., May 2, 1903, p. 1210) has observed 'that irritation, which may arise from irritation of mucous membranes and affects the pneumogastric and other nerve-filaments, is strikingly observed when the nasal mucous membrane is irritated, and may cause an inhibition of the heart action.' If this be true, can we not, by obtunding the sensibility of the nasal mucous membrane and thereby removing this source of reflex irritation, not only greatly reduce the frequency and severity of the vomiting but also the danger of cardiac and respiratory failure as well as the shock resulting from operation?"

In 1904 I anesthetized a patient who had a weak heart, and who had been told by a number of physicians that she could not safely take chloroform. To fortify the heart, I gave her, before the operation, 8 drops of adnephrin solution (1 : 1000) and also painted the nasal cavities clear back to the throat with cocaine—i. e. 10 grains dissolved in 1 ounce of a 1 : 4000 solution of the adnephrin. She took the anesthetic very nicely, without any heart failure, weakness or shock of any kind, and made a prompt recovery.

Since that time I have always employed this same preparation in those cases in which I administer chloroform. I have given it in more than 100 cases since that time, and I have never seen any bad result; there have been only a few instances of vomiting, and recovery has been prompt and satisfactory.

In some cases a one-percent solution of cocaine has been used. Anyone who will use this solution, as just described, and will apply it thoroughly, noting how it dilates the nasal passages, how much more freely the patient breathes after its use, and how much better the general condition is with it than without its use, will not fail to employ it after that.

While I have not been able to give the specific reasons for this reduction of shock and general well-being following operations, I have been firmly convinced that these are facts, and have on numerous occasions called attention to them at medical-society meetings and elsewhere. While some physicians have manifested considerable interest in the method, others have received it with that supercilious toleration which some so-called "great men" are prone to bestow upon any unusual suggestion.

In view of the facts here set forth, it affords me a great deal of satisfaction to call attention to a paper read before the Academy of Medicine of Paris (see *Journal A. M. A.*, p. 1211, April 20, 1912) on March 5, 1912. In this paper, prepared conjointly with Drs. Herrinschmidt and Beauvy, Prof. Delbet shows that chloroform produces pronounced changes in the suprarenal glands, changes of which the articles in *The Journal of the American Medical Association* speaks as follows:

"Without intending to attribute all the sequels of chloroform anesthesia to alterations of the suprarenal glands, Dr. Delbet is thoroughly convinced that certain of these sequels are due to these alterations, and that it is possible to diminish or avoid them by reinforcing the insufficient suprarenal function by injections of epinephrin or of extract of suprarenal glands. There are, indeed certain sequels which are entirely attributable to suprarenal insufficiency. Operative shock is one of these. It is characterized essentially by asthenia and weakness of the pulse, which are the symptoms of suprarenal insufficiency."

For ordinary operations Dr. Delbet injects 0.4 mg. (about 6 minims 1 : 1000 adrenaline or similar solution) before the operation, and in severe operations he uses 0.6 mg. suprarenalin, at one dose, or, if needed, repeats the doses until he has used 1 mg. in twenty-four hours. According to his experience with more than 1000 patients, epinephrin administered subcutaneously diminishes, and, in the majority of cases, entirely suppresses operative shock.

I am convinced that a free application of my solution to the nose, clear back to the pharynx, acts in the same way but probably does not have such a pronounced effect. I shall be pleased to have physicians try my method and report results to me.—*Clinical Medicine.*

